# Different approaches for delivery of Intermittent Preventive Treatment (IPT) to pregnant women in Burkina Faso

**DOI:** 10.1186/1475-2875-9-S2-P33

**Published:** 2010-10-20

**Authors:** Alphonse Ouédraogo, Sheick O Coulibaly, Amidou Diarra, Abdoulaye Traoré, Sodiomon B Sirima, Pascal Magnussen

**Affiliations:** 1 Centre National de Recherche et de Formation sur le Paludisme, Ouagadougou, Burkina Faso; 2 Laboratoire National de Santé Publique, Ouagadougou, Burkina Faso; 3 Université de Ouagadougou, Burkina Faso; 4 Groupe de Recherche Action en Santé, Ouagadougou, Burkina Faso; 5 Université de Copenhague, Denmark

## Background

In Africa the burden of malaria in pregnancy is highest in rural areas. In many Sub-Saharan African countries, IPTp/SP is being adopted to replace chloroquine (CQ) chemoprophylaxis shown to be inefficacious. Many publications showed a very poor compliance with CQ chemoprophylaxis among pregnant women and might explain the failure of this preventive strategy rather than increased levels of CQ resistance. In this study, we compare three approaches of IPTp/SP delivery to pregnant women in term of improving coverage and compliance. These three approaches are: i) Passive health centre services, ii) Extended delivery outreach services, iii) Community based distribution delivery approach.

## Materials and methods

The study was taking place in the health district of Saponé. A total of 12/14 sub-districts were randomly selected. Each community clinic and its catchments areas were considered as a cluster. Clusters were also randomly assigned to 2 interventions and 1 control arms; 4 clusters were assigned to each arm. Two cross sectional surveys were planned to measure key outcome indicators; one at the beginning (pre-intervention) of the trial and the second one at the end of the study (post-intervention). Clinical & biological data were collected (parasitemia & haemoglobin).

## Results

**Figure 1:**
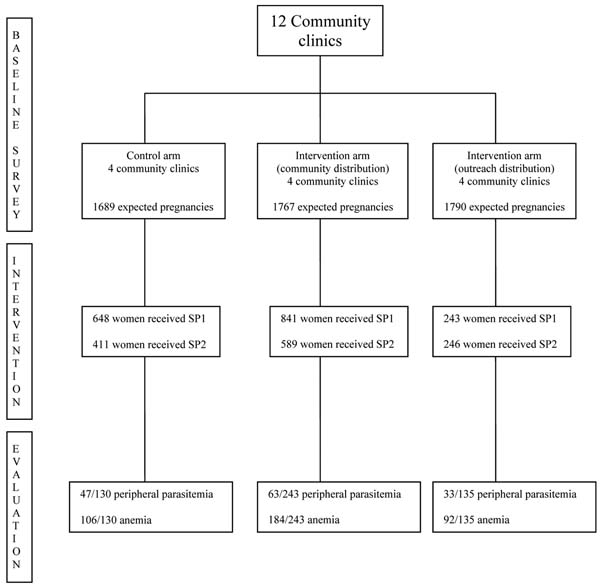
Study profile.

The mean coverage of 2 doses of IPT is higher in the community based arm than in the control group (33% vs 24%; P<0.001). The compliance was better in the control group than intervention groups (P=0.001). After the intervention, there was decrease of peripheral parasitemia from 32.2% at health units to 25.9% at community based approach (P=0.03). There was also slightly decrease of anemia from 68.1% at outreach distribution approach compared to health units 81.5% (P=0.01). (see figure 1)

## Conclusions

Therefore a combination of health facility-based and community-based approaches might be needed to maximise the impact of IPTp.

